# Hypoglycaemia related to inherited metabolic diseases in adults

**DOI:** 10.1186/1750-1172-7-26

**Published:** 2012-05-15

**Authors:** Claire Douillard, Karine Mention, Dries Dobbelaere, Jean-Louis Wemeau, Jean-Marie Saudubray, Marie-Christine Vantyghem

**Affiliations:** 1 Service d’Endocrinologie et maladies Métaboliques, Hôpital Claude Huriez, Centre Hospitalier Régional et Universitaire de Lille, 1, Rue Polonovski, Lille cedex, 59037, France; 2 Centre de Référence des Erreurs Innées du Métabolisme –Hôpital Jeanne de Flandres, Centre Hospitalier Régional et Universitaire de Lille, avenue Eugène Avinée, Lille cedex, 59037, France; 3 Departement des maladies métaboliques, Fédération des maladies du système nerveux, Hôpital Pitié-Salpêtrière, 47-83 Boulevard de l’Hôpital, 75651, Paris cedex 13, France

**Keywords:** Inborn errors of metabolism, Hypoglycaemia, Non-insulinoma pancreatogenic hypoglycaemia syndrome, Glycogen storage disease, Fatty acid oxidation disorder, Gluconeogenesis

## Abstract

In non-diabetic adult patients, hypoglycaemia may be related to drugs, critical illness, cortisol or glucagon insufficiency, non-islet cell tumour, insulinoma, or it may be surreptitious. Nevertheless, some hypoglycaemic episodes remain unexplained, and inborn errors of metabolism (IEM) should be considered, particularly in cases of multisystemic involvement. In children, IEM are considered a differential diagnosis in cases of hypoglycaemia. In adulthood, IEM-related hypoglycaemia can persist in a previously diagnosed childhood disease. Hypoglycaemia may sometimes be a presenting sign of the IEM. Short stature, hepatomegaly, hypogonadism, dysmorphia or muscular symptoms are signs suggestive of IEM-related hypoglycaemia. In both adults and children, hypoglycaemia can be clinically classified according to its timing. *Postprandial* hypoglycaemia can be an indicator of either endogenous hyperinsulinism linked to non-insulinoma pancreatogenic hypoglycaemia syndrome (NIPHS, unknown incidence in adults) or very rarely, inherited fructose intolerance. Glucokinase-activating mutations (one family) are the only genetic disorder responsible for NIPH in adults that has been clearly identified so far. *Exercise-induced* hyperinsulinism is linked to an activating mutation of the monocarboxylate transporter 1 (one family). *Fasting* hypoglycaemia may be caused by IEM that were already diagnosed in childhood and persist into adulthood: glycogen storage disease (GSD) type I, III, 0, VI and IX; glucose transporter 2 deficiency; fatty acid oxidation; ketogenesis disorders; and gluconeogenesis disorders. Fasting hypoglycaemia in adulthood can also be a rare presenting sign of an IEM, especially in GSD type III, fatty acid oxidation [medium-chain acyl-CoA dehydrogenase (MCAD), ketogenesis disorders (3-hydroxy-3-methyl-glutaryl-CoA (HMG-CoA) lyase deficiency, and gluconeogenesis disorders (fructose-1,6-biphosphatase deficiency)].

## Introduction

Inborn errors of metabolism (IEM) are inherited diseases, usually recessive, which have recently become an important unrecognized part of adult medicine. They are usually classified into 3 main groups: 1) intoxication diseases (i.e., amino-acidopathies, organic aciduria, fructose intolerance and galactosaemia, iron and copper overload, porphyria); 2) diseases linked to energy deficiency (i.e., glycogenolysis, mitochondrial diseases, disorders of fatty acid oxidation and ketogenesis, congenital lactic acidosis); and 3) diseases due to degradation or synthesis defect of complex molecules (i.e., lysosomal or peroxisomal diseases, and congenital disorders of glycosylation). The clinical presentations of these disorders are very diverse and can encompass any symptoms at any age in any scenario with any mode of inheritance [1]. The aim of this paper is to focus on the diagnostic approach to these metabolic disorders in adults presenting with hypoglycaemia.

The diagnosis of hypoglycaemia in adults must first be firmly established before starting a diagnostic work-up that includes numerous and costly investigations. A positive diagnosis of hypoglycaemia is made when the venous blood glucose level is <0.55 g/L (or <3 mmol/L), obtained if possible at the time of the symptoms [2].

**Figure 1:**
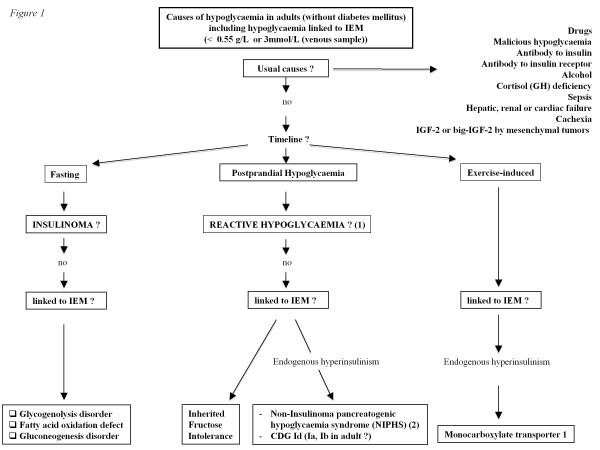
**Causes of hypoglycaemia in adults (without diabetes mellitus) including hypoglycaemia linked to IEM.** (1) Dysregulation of insulin secretion; Increase of insulin sensitivity; Dumping syndrome; Endogenous hyperinsulinism post surgery (gastric by-pass). (2) NIPHS (Non-Insulinoma pancreatogenic hypoglycaemia syndrome): Glucokinase activating mutation (and SUR1/Kir6.2, SCHAD, GDH mutations in adult ?).

In a non-diabetic adult patient, hypoglycaemia may be related to drugs, critical illness, cortisol or glucagon insufficiency, non-islet cell tumour, insulinoma, or it may be surreptitious (Figure 1) [2]. Some hypoglycaemic episodes remain unexplained, in which case the differential diagnosis should be narrowed down to less frequent causes such as inherited metabolic diseases, especially when multisystem involvement is present.

The steps for obtaining a diagnosis do not differ from those of the usual work-up, i.e., identification of the elapsed time from the last meal, circumstances of occurrence (type of meal, catabolism, concomitant infection, and physical activity), and use of drugs (type and dose). Careful physical assessment is very important, as short stature, hepatomegaly, hypogonadism, dysmorphia or muscular symptoms may suggest IEM-related hypoglycaemia.

In children presenting with hypoglycaemia, IEM appear consistently in the differential diagnosis. In adulthood, IEM-related hypoglycaemia can persist in a previously diagnosed childhood disease. In this case, hypoglycaemic episodes are usually less severe and occur less frequently than in childhood. Although rare, hypoglycaemia may be a presenting sign of IEM, even in adulthood, and must not be overlooked.

## Diagnosis

The diagnostic strategy in cases of hypoglycaemia, including those linked to an IEM without malformative syndrome, is analysed in Figure 1. Basically, true hypoglycaemia must be identified before the diagnostic work-up is started [2]. The main issue is that the clinical signs of hypoglycaemia can lack specificity, explaining why the diagnosis of “hypoglycaemia” is often wrongly made from clinical symptoms that are independent of true hypoglycaemia. A positive diagnosis of hypoglycaemia is made when the venous blood glucose level is <0.55 g/L (or <3 mmol/L), obtained if possible at the time of the symptoms. If a blood glucose level is not available, repeated capillary glucose measurements <0.55 g/L that are not performed by the patient himself should incite the diagnostic work-up for hypoglycaemia. The presence of the well known Whipple’s triad is therefore mandatory for starting this workup. The Whipple’s triad is considered positive if biochemical hypoglycaemia (blood glucose level <0.55 g/L) is accompanied by hypoglycaemic symptoms that resolve after correction of the hypoglycaemia [2].

There are several characteristic symptoms oh hypoglycaemia. First, these symptoms are varied and non-specific. Hypoglycaemia stimulates the autonomic nervous system with an adrenergic (pallor, tremor, anxiety, arterial hypertension) and cholinergic (sweat, hunger, paresthesias) reaction, which is similar to the reaction observed in anxiety. Second, neuroglycopaenic symptoms can occur and are explained by a low glucose level in the central nervous system. These symptoms are highly suggestive of a hypoglycaemia diagnosis, starting with cortical symptoms (drowsiness, confusion, visual impairment) and, if hypoglycaemia is severe, continuing to motor deficit, seizure and loss of consciousness.

In both adults and children, IEM-related hypoglycaemia can be clinically classified according to its timing as *postprandial*, *exercise-induced* or *fasting* hypoglycaemia. *Postprandial* hypoglycaemia can be an indicator of either endogenous hyperinsulinaemia linked to a non-insulinoma pancreatogenic hypoglycaemia syndrome or very rarely, inherited fructose intolerance. *Exercise-induced* hyperinsulinaemia is linked to an activating mutation of monocarboxylate transporter 1 transmitted as an autosomal dominant trait. *Fasting* hypoglycaemia may be caused by an IEM that was already diagnosed in childhood and persists into adulthood, and may sometimes even be a presenting sign of the disease. These IEM include liver glycogen storage disease, fatty acid oxidation and ketogenesis defects, and gluconeogenesis disorders. The laboratory tests usually requested by an endocrinologist and including those that are more specifically oriented toward an IEM (in italics) are :

Plasma glucose, insulin, C-peptide

Creatinine, liver parameters, NF, CRP, CPK, uric acid, triglycerides

Cortisol, GH, IGF1

Oral hypoglycaemic agents

Blood lactate

Free fatty acids

3-ßOH butyrate

Acylcarnitines (dried blood spots)

Plasma amino acids

Ammonaemia

Carnitine status (free and total)

Urine dipstick (for ketone bodies)

Urinary organic acids

It is of great importance that these tests be performed on blood and urine samples during the acute hypoglycaemic episode. Tables 1 and 2 show some clinical and biological features suggestive of specific IEMs.

**Table 1:** Signs of an IEM

**When to consider a glycogenolysis disorder**	**When to consider a fatty acid oxidation disorder**	**When to consider a gluconeogenesis disorder**
**-** Fasting hypoglycaemia	- Fasting hypoglycaemia	- Long fasting hypoglycaemia
- Presence of ketosis (except for GSD I)	- Absence of ketosis	- Presence of ketosis
- High blood lactate	- High CPK levels	- Lactic acidosis
- Hyperuricaemia	- Acylcarnitine accumulation (*example: MCAD: high C8 and high C8/C2 ratio)*	- High alanine level during fasting
**-** Hypertriglyceridaemia	- Urine organic acid accumulation (*example*: *HMG-CoA lyase: 3-Hydroxy-3-Methylglutaric acid)*	- Blood glycerol and urine glycerol-3-phosphate accumulation during fasting
- Hepatomegaly	- High free carnitine plasma level (CPT1)	
- Muscular signs	- Family history of sudden death	

**Table 2:** Main causes of hypoglycaemia related to inborn errors of metabolism (IEM) in adults

**Diseases**	**Persistent hypoglycaemia**	**Hypoglycaemia as an indicator**	**Positive and molecular diagnosis**
**Fasting hypoglycaemia**			
**Glycogenolysis disorders**			I: high blood lactates especially before meals, hypertriglyceridaemia, hyperuricaemia
*Glycogen Storage Disease*			Ketones rather low
Liver:			
I, VI, IX, 0, Fanconi-Bickel			III: high blood lactates especially after meals, hypertriglyceridaemia,
	I, III, 0, Fanconi-Bickel		Ketones rather high
Mixed:			
III, IV		III (debranching enzyme or amylo-1,6-glucosidase enzyme)	GSD I/III: DNA (leucocytes)
**Defect of fatty acid oxidation**			- no ketones (or rather low during hypoglycaemia)
- carnitine cycle (CPT1/2)	- CPT1		- high free carnitine plasma level (CPT1)
- ß fatty acid oxidation	- VLCAD, MCAD, SCHAD, LCHAD		- accumulation of plasma acylcarnitines (example : *High C8 and high C8/C2 ratio in MCAD*)
- electron transfer			
- ketogenesis	- HMG-CoA lyase	MCAD	- urine organic acid accumulation - ß oxidation *in vitro* (lymphocytes)
			- DNA (leucocytes)
**Gluconeogenesis**	Fructose-1,6-biphosphatase	Fructose-1,6-biphosphatase	- lactic acidosis
			- high alanine
			- ketosis
			- high glycerol
			- urine: high 3 Ph glycerol
**Postprandial hypoglycaemia**			
NIPHS (sometimes in fasting period)	- hypoglycaemia during infancy (SUR.1, Kir6.2, SCHAD, GDH, Glucokinase	- Glucokinase mutation	- hyperinsulinism
	- sometimes progression to diabetes in adulthood (especially SUR-1)		- hyperammonemia (GDH) - Genotyping (DNA leucocytes)
CDG Ia, Ib, Id (sometimes at anytime)	- CDG Id	no	Isoelectric focussing of transferrin
			- phosphomannomutase gene (Ia)
			- phosphomannomutase isomerase gene (Ib)
			- 1,3-mannosyltransferase (Id)
			Hyperinsulinism
Inherited fructose intolerance	- After ingestion of fructose (fruits, sucrose, sweet foods)		- fructose test tolerance iv
	- Late postprandial period		- breath test fructose
			- DNA leucocytes : aldolase B gene
**Exercise-induced hypoglycaemia**			
NIPHS	- MCT1	yes	Genotyping (DNA leucocytes)

GSD, glycogen storage disease; CPT, carnitine palmitoyltransferase-1; VLCAD, very long-chain acyl-CoA dehydrogenase; MCAD, medium-chain acyl-CoA dehydrogenase; LCHAD, long-chain 3-hydroxyacyl-CoA dehydrogenase; SCHAD, short-chain L-3-hydroxyacyl-CoA dehydrogenase;

lGDH, glutamate dehydrogenase; MCT1, monocarboxylate transporter 1; CDG, congenital disorders of glycosylation; NIPHS, non-insulinoma pancreatogenic hypoglycaemia syndrome.

The diagnostic work-up for hypoglycaemia is presented in the section with the same heading and describes the different causes of IEM-related hypoglycaemia.

## Postprandial hypoglycaemia

### Endogenous hyperinsulinaemia

#### Non-insulinoma pancreatogenic hypoglycaemia syndrome (NIPHS)

Postprandial hypoglycaemia is usually related to endogenous hyperinsulinaemia, or non-insulinoma pancreatogenic hypoglycaemia syndrome (NIPHS). NIPHS in adulthood was called “nesidioblastosis” in the past and is characterized, as in childhood, by an increase in the number and volume of beta cells. Three to five percent of all causes of hyperinsulinaemia are related to NIPHS diagnosed in adulthood [3-5]. Hyperinsulinaemia is a heterogeneous disorder that can be caused by various defects in the regulation of insulin secretion by the pancreatic β-cells [6]. In children these include 1) channelopathies affecting either the SUR 1 (ORPHA79643) [7] or the Kir channel (ORPHA79644) [8]; 2) enzyme defects involving glucokinase [9], glutamate dehydrogenase [10], or short-chain L-3-hydroxyacyl-CoA dehydrogenase (SCHAD) [11]; and 3) defects of the monocarboxylate transporter 1 (MCT1) [12] and the mitochondrial uncoupling protein 2 (UCP2) [13]. These metabolic diseases disturb ß-cell intramitochondrial energy metabolism and induce non-regulated ATP overproduction, which causes abnormal inactivation of the potassium channel and stimulation of the calcium channel responsible for hyperinsulinaemia. In addition, modifications of insulin secretion implicating the insulin receptor [14] and, more recently, Hepatocyte Nuclear Factor 4 (HNF4) alpha, a transcription factor involved in maturity-onset diabetes of the young (MODY), have also been described (ORPHA263455) [15]. Up till now, in endogenous hyperinsulinism, neither inactivating mutations of SUR 1, Kir channel and SCHAD (ORPHA35123) [11] nor activating mutations of glutamate dehydrogenase (GDH, Hyperinsulinism-Hyperammonaemia syndrome, ORPHA35878) [16,17] have been observed in adulthood [18]. This is possibly due to an early presentation of these disorders in children, which are not seen in adulthood. However the possibility that such rare metabolic mechanisms are overlooked and are not yet familiar to adult endocrinologists cannot be excluded. Finally, glucokinase-activating mutation transmitted as an autosomal dominant trait is the only genetic disorder that has been clearly identified in adults to date (<1-9/1,000,000; ORPHA79299) [19].

From a clinical point of view, all of the causes of hyperinsulinism listed above (except monocarboxylate transporter 1, see below) actually present mainly with postprandial hypoglycaemia but can also appear in the fasting state.

#### Congenital disorders of glycosylation (CDG)

CDG syndromes (<1-9/100,000, ORPHA137), especially the Ia (ORPHA79318), Ib (ORPHA79319) and Id (ORPHA79321) types, can also lead to hypoglycaemia via hyperinsulinaemia [20-22]. A few cases have been reported in adults, particularly a case of CDG-Id in a patient who died at the age of 19 years from hyperinsulinaemic hypoglycaemia, with β-cell hyperplasia on the autopsy, associated with Dandy-Walker malformation, dysmorphic facial features, and marked hypotonia. A unique homozygote mutation of the *ALG3* gene encoding mannosyltransferase was identified [23].

### Inherited fructose intolerance

Inherited fructose intolerance (HFI or fructosaemia, <1-9/100,000; ORPHA469) is linked to a fructose-1-phosphate aldolase deficiency. In this autosomal recessive disorder, fructose ingestion (especially that contained in fruits, but also sucrose, which is found in many sweet foods) generally induces gastrointestinal disorders and postprandial hypoglycaemia. In case of misdiagnosis, it leads to hepatomegaly and proximal tubular dysfunction, sometimes with liver steatosis and kidney failure with delayed growth.

This disease may manifest in adulthood and should be considered in case of hypoglycaemia with digestive intolerance in relation to any intake of fructose, and aversion to sweet food [24]. The diagnosis was made at the age of 50 in a woman who presented a life-long history of aversion to sugary foods (nausea, vomiting, diffuse abdominal pain and hypoglycaemic symptoms, even after the smallest amount of sugar or fruit) [25]. Some other adult cases were diagnosed after the development of life-threatening reactions from intravenous infusions containing fructose, sorbitol or invert sugar (a mixture of glucose and fructose obtained by hydrolysis of sucrose) when these intravenous solutions were still in use [26]. Because approximately half of all adults with HFI are free of dental caries, the diagnosis has also been made by dentists. Although several hundred patients with HFI have been identified since its recognition as an inborn error of metabolism in 1957, these observations indicate that affected subjects may remain undiagnosed and still have a normal life span.

Whenever HFI is suspected, fructose should be eliminated. The beneficial clinical and chemical effects of withdrawal, usually seen within days, provide the first diagnostic clue. If the nutritional history is suggestive, or if other aspects are indicative of HFI (e.g.*,* a positive family history), the disorder should be confirmed by molecular diagnosis on DNA from peripheral leukocytes. This is a non-invasive approach and has an advantage over enzymatic measurement in liver tissue since it eliminates the complication of secondary lowered aldolase activity in a damaged liver. The three most common mutations are responsible for more than 90% of HFI cases in some European regions and for still more than 50% of cases from the more heterogeneous population in North America [http://www.bu.edu/aldolase/HFI/hfidb/DistribTable.htm].

The long-term course is usually considered favourable despite a risk of vitamin C deficiency requiring supplementation [27].

## Exercise-induced hypoglycaemia

Exercise-induced hypoglycaemia, also caused by hyperinsulinaemia, has been described both in childhood and adulthood and is due to failed silencing of monocarboxylate transporter 1 in pancreatic β cells, which is coded by the SLC16A1 gene [12]. This mutation causes an over-transportation of pyruvate into cells, which in pancreatic beta cells results in an unregulated overproduction of ATP. Hypoglycaemia occurs only after physical exercise and can be triggered by an IV infusion of pyruvate. It is an autosomal dominant transmitted trait (< 1-9/1,000,000).

## Fasting hypoglycaemia

In adulthood, IEM that induce fasting hypoglycaemia and that were diagnosed in childhood are usually less severe and less frequent, even if type I glycogen storage diseases can lead to severe hypoglycaemia in adults. Otherwise, fasting hypoglycaemia in adulthood may be a presenting sign of an IEM, although they are rare.

### Glycogenolysis disorders

In childhood, glycogen storage diseases (GSD), especially the liver and mixed types, are classical causes of short-term fasting hypoglycaemia (few hours) (See Table 2). Some of these GSD, particularly types I (annual incidence around 1/100,000, ORPHA364), III (<1-9/100,000, ORPHA366), 0 (<1-9/1,000,000, ORPHA2089) [28] and Fanconi-Bickel syndrome due to Glut 2 deficiency (<1-9/1,000,000, ORPHA2088) [29], can induce hypoglycaemia that persists into adulthood. Type III GSD in adulthood is the only one that has been diagnosed in a hypoglycaemia work-up; this was in a 47-year-old woman presenting with a history of fasting hypoglycaemia and muscle weakness since childhood [30]. Note that GSD (for example, type I) can also be diagnosed in adulthood during the investigation for liver adenomatosis or myopathy.

### Defects of fatty acid oxidation (FAO)

This common group of autosomal recessive disorders is classified into 4 sub-groups: 1) carnitine cycle anomalies; 2) long-, medium- and short-chain fatty acid ß-oxidation disorders; 3) electron transfer disturbances (type II glutaric aciduria or multiple defect in acyl-CoA dehydrogenase, ORPHA26791); and 4) anomalies of ketone body synthesis [31]. Clinical symptoms are essentially similar amongst the four groups. These disorders are found either in the neonatal period, or later during childhood, adolescence or even in adulthood, often during a concomitant disease [32]. They may sometimes have a very long asymptomatic phase, thus making the diagnosis difficult. Clinical signs vary according to age. In neonates, non-ketotic hypoglycaemia is associated with hyperammonaemia, mild metabolic acidosis, along with organic aciduria, cardiomyopathy, rhabdomyolysis, liver signs and Reye’s syndrome. Myopathy is the main manifestation in adulthood, but hypoglycaemia can persist or be an indicator.

1- Defects in the carnitine cycle are characterized by non-ketotic hypoglycaemia, hyperammonaemic encephalopathy, cardiomyopathy, myopathy or liver disease, usually occurring in childhood. Blood carnitine levels are very low (except for defects in carnitine palmitoyltransferase-1), and the diagnosis can be confirmed on fibroblast cultures or on lymphocytes [33]. Carnitine transfers long-chain fatty acids into the mitochondria, making them available for ß-oxidation and ketogenesis by means of enzymes and transporters such as OCTN2 carnitine transporter (involved in primary carnitine deficiency, ORPHA158), carnitine palmitoyltransferase-1 (CPT1, <1/1,000,000; ORPHA156), carnitine/acylcarnitine translocase (CACT, <1/1,000,000; ORPHA159), and carnitine palmitoyltransferase-2 (CPT2, <1/1,000,000; ORPHA157). In childhood, hypoglycaemia is seen in CPT1 and CPT2 deficiency and can persist into adulthood (especially CPT-1), although it is less frequent and less severe [34]. Hypoglycaemia has not been a presenting sign in any cases linked to this disorder in adulthood.

2- With regard to fatty acid ß-oxidation disorders, hypoglycaemia can be an indicator of VLCAD (very long-chain acyl-coA dehydrogenase, ORPHA26793), MCAD (medium-chain acyl-coA dehydrogenase, < 1-5/10,000, ORPHA42), LCHAD (long-chain 3-hydroxyacyl-coA dehydrogenase, <1/1,000,000; ORPHA5) and SCHAD (short-chain 3-hydroxyacyl-coA dehydrogenase; ORPHA35123) in childhood, which can sometimes persist into adulthood. SCHAD deficiency induces hyperinsulinemic hypoglycaemia, the mechanism of which has been recently identified [16] (see above). To our knowledge, MCAD is the only ß-oxidation disorder in which hypoglycaemia has led to a diagnosis in adulthood. A case of MCAD defect was reported in a 33-year old man hospitalized for headaches, vomiting, confusion, coma, respiratory alkalosis with hypoglycaemia, hyperlactacidaemia, hyperammonaemia, and ventricular tachycardia, followed by a cardiac arrest with renal insufficiency and rhabdomyolysis [35]. MCAD was diagnosed in two men (29 and 32-years-old) who had presented since childhood with mild hypoglycaemic symptoms and had a family history of MCAD [36,37]. As for other fatty acid ß-oxidation disorders, some of them (VLCAD, SCHAD) can be diagnosed in adulthood, but the presenting symptoms are muscular [38-41]. Regardless of age, cardiac arrest or sudden infant death can occur and should suggest the diagnosis. In pregnant women, intrauterine growth restriction, prematurity, preeclampsia and acute liver steatosis of pregnancy or haemolytic anaemia, elevated liver enzymes and low platelet count (HELLP) -syndrome can occur in heterozygous mothers if the fœtus bears a defect in the LCHAD, trifunctional protein or carnitine palmityltransferase [42-45]. The clinical presentation of homozygous patients with LCHAD includes myopathy, recurrent episodes of rhabdomyolysis, arrhythmia, cardiomyopathy and neuropathy, as well as pigmentary retinitis with possible blindness [45].

3- Electron transfer disturbances (glutaric aciduria type II or multiple acyl-CoA dehydrogenase deficiency, ORPHA26791) involve severe manifestations in the neonatal period: hypoglycaemia, acidosis, and cardiomyopathy, sometimes associated with congenital malformations (kidney, brain, facial dysmorphism). There have been no reports of partial deficiencies with hypoglycaemia manifestations in the literature in adulthood. The oldest age for disease onset is 14-years-old in a Chinese adolescent boy who presented with severe vomiting, followed by rapid deterioration leading to death. However, in this case, hypoglycaemia was not a presenting symptom.

4- Ketogenesis disorders [defect in 3-hydroxy-3-methyl-glutaryl-CoA (HMG-CoA) synthase (<1-9/1,000,000, ORPHA35701) and HMG-CoA lyase (ORPHA20) are indicated most of the time by acute fasting hypoketotic hypoglycaemia during the neonatal or sometimes infancy period. The diagnosis might however be delayed, as was the case for a 36-year-old woman who had a history of hypoglycaemia and seizures during childhood but was finally diagnosed only at the age of 36 years after a new severe aketotic hypoglycaemic episode below 3 mmol/L with neurological symptoms (seizures, leucodystrophy) [31]. The diagnosis was an HMG-CoA lyase defect (See Table 2).

The diagnosis of all these FAO disorders relies on urine organic acid and plasma acylcarnitine profiles during hypoglycaemia, and the simple quick urine dipstick test for ketone bodies is a strong indicator if negative with concomitant hypoglycaemia.

### Gluconeogenesis disorder

During childhood, defects of fructose-1,6-biphosphatase (<1-9/100 000, ORPHA348) lead to recurrent attacks of fasting hypoglycaemia and lactic acidosis triggered by fasting and catabolic circumstances. In adulthood, a defect in fructose-1,6-biphosphatase was diagnosed in a young woman who presented during the second trimester of pregnancy with recurrent hypoglycaemia and acidosis [46]. Pregnancy is possible: 3 cases have been reported in the same patient who had followed a strict diet with nocturnal enteral nutrition [45]. These pregnancies were complicated by mild gestational diabetes, increased need for uncooked cornstarch and postpartum hypoglycaemia. No complications were noted in the children. Nevertheless, the mother developed deafness and cognitive impairment afterwards.

## Other rare cases of hypoglycemia linked to iem but not yet described in adults

### Organic aciduria

Different types of organic aciduria can occasionally induce hypoglycaemia, especially during acute metabolic decompensation. Type II glutaric aciduria or multiple defects in acyl-CoA dehydrogenase (ORPHA26791) have already been mentioned. Severe hypoglycaemia has been reported only in a few cases, usually early in life. These defects include 3 methylcrotonyl-coenzyme A carboxylase [47,48], gammahydroxybutyric aciduria, and defects in malonyl-coenzyme A decarboxylase (a rare disorder associated with metabolic acidosis, hypoglycaemia and/or cardiomyopathy and neurological signs) [49].

### Biotin-responsive multiple carboxylase deficiency

Some cases of defects in biotinidase (BRMCD: biotin responsive multiple carboxylase deficiency) are generally associated with episodes of coma, lactic acidosis with ketosis, neurological disorders and cutaneous symptoms (rashes, eczema, alopecia) in patients aged 3 months to 14 years [50,51]. A few cases have been associated with hyperglycaemia, which may suggest diabetes due to the ketoacidosis. In contrast, hypoglycaemia is sometimes present. The administration of biotin 10 mg/day provides a dramatic and sustained improvement.

### Respiratory chain defects

Respiratory chain defects can also lead to hypoglycaemia during the neonatal period or in early infancy to childhood [52].

## Diagnosis workup

**Figure 2:**
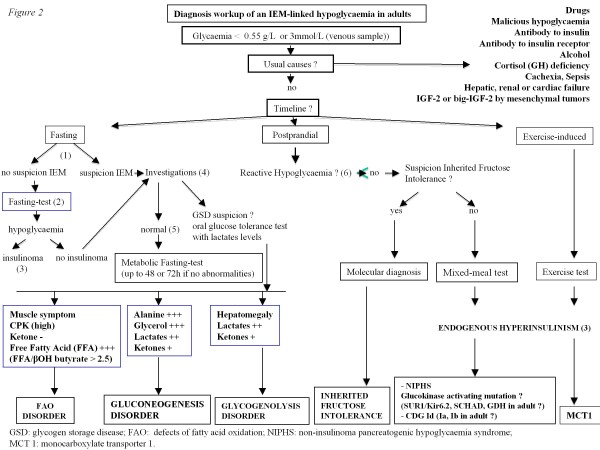
**Diagnosis workup of an IEM-linked hypoglycaemia in adults.** (1) suspicion IEM: familial history of hypoglycaemia or rhabdomyolysis, hypoglycaemia starting during childhood, unexplained muscular symptoms (myalgias, high CPK levels), cardiac involvement and/or hepatomegaly. (2) fasting of up to 72 h if no abnormalities. Blood glucose, cortisol, Growth Hormone, insulin, C peptide, proinsulin and beta hydroxybutyrate should be measured every 6 h if the blood glucose level is > 0.6 g/L and then every 1 to 2 h (with capillary blood glucose test) if this level drops below 0.6 g/L. Fasting should be stopped if the blood glucose level is <0.45 g/L and occurs with hypoglycaemia symptoms, or if this level is <0.3 g/L regardless of the symptoms. Baseline blood measurements should be performed before glucose intake. (3) if glycaemia < 0.55 g/L (3 mmol/L): Detectable insulin (> 3 mIU/L (18 pmol/L), IRMA), C peptide > 0.6 ng/ml (0.2 nmol/l), β-OH butyrate < 2.7 mmol/L, sulfamide and repaglinide negative, 1 mg Glucagon IV test : + 0.5 g/L at 30 min (1.4 mmol/L). (4) investigations: abdominal ultrasound, EKG, echocardiography and laboratory measurements including acylcarnitine profile, total and free carnitin, urine organic acids, CPK, blood lactate levels, triglycerides, uric acid, liver enzymes. (5) normal or no evidence for defects of FAO. (6) CGMS (Continuous Glucose Monitoring System).

When true hypoglycaemia, according to the above definition and meeting the Whipple’s triad criteria, is diagnosed, the usual causes (as presented in the introduction section and in Figure 1) should first be ruled out. The diagnosis workup is summarized in Figure 2.

### Postprandial hypoglycaemia

Postprandial symptoms are non-specific and are usually due to an adrenergic reaction. If these symptoms are not associated with true hypoglycaemia, the term “postprandial syndrome” is preferred. If the blood glucose level is <0.55 g/L, then the term reactive (or functional) hypoglycaemia (RH) should be used [53]. CGMS (continuous glucose monitoring system) can help to diagnose RH. The oral glucose tolerance test is difficult to interpret, as moderate biological hypoglycaemia is frequent but is not systematically associated with clinical symptoms. RH can be due to:

a. insulin secretion impairment, with excessive insulin secretion in response to food intake containing carbohydrates with a high glycaemic index;

b. increased insulin sensitivity in young and slim patients [54];

c. hypoglycaemia in patients with gastrectomy with or without pyloroplasty or vagotomy, leading to dumping syndrome (abdominal pain, nausea, diarrhoea and hypoglycaemia due to excessive insulin secretion in response to an excessive intake of carbohydrates);

d. endogenous hyperinsulinism after Roux-en-Y gastric bypass for obesity, first described by GJ Service et al. [55], in which GLP-1 plays a role.

Lastly, if none of these causes can explain postprandial hypoglycaemia, then an IEM could be hypothesized.

a. If the clinical context is suggestive of HFI, an assessment should be done for hepatomegaly and liver enzyme abnormalities. If the diagnosis is highly probable, and even if the liver enzymes are normal, the molecular diagnosis of HFI should be performed.

b. If the clinical context is not suggestive of HFI, then endogenous hyperinsulinism should be ruled out (Figure 2). The recent guidelines suggest that a mixed meal (recreating if possible the circumstances of hypoglycaemia) is indicated with the following measurements done at baseline and every 30 min for 5 h: blood glucose level, insulin, C-peptide, proinsulin, beta hydroxybutyrate, sulfamide and repaglinide. If hypoglycaemia occurs (neuroglycopaenic symptoms and blood glucose level <0.45 g/L), the test should be stopped but with a last measurement taken before glucose intake. If endogenous hyperinsulinism is present, an investigation for insulinoma should be done, as there have been rare observations of insulinoma presenting with postprandial hypoglycaemia. Therefore, a CT scan, MRI, endoscopic ultrasound, octreoscan and PET scan are mandatory. If the results are negative or if there are discrepancies, a work-up for some types of mutations can be done: glucokinase activation mutation, mainly if there is a family history (autosomal dominant transmission), SUR-1 or Kir 6–2, because these mutations are frequent in children but have not yet been described in adults. If there are neurological symptoms with hyperammonaemia, investigations can be done for GDH mutations (though not yet described in adults) and SCHAD. Lastly, if the clinical symptoms are suggestive (malformations, facial dysmorphy, hypogonadism), type Id CDG should be ruled out.

### Exercise-induced hypoglycaemia

If exercise-induced hypoglycaemia is suspected or diagnosed, an exercise test should be performed to recreate the circumstances in which symptomatic hypoglycaemia is likely to occur. If endogenous hyperinsulinism is diagnosed, an investigation for SLC16A1 (coding for monocarboxylate transporter 1) mutation should be done.

### Fasting hypoglycaemia

The presence of fasting hypoglycaemia should prompt practitioners to look for a clinical context compatible with IEM: family history of hypoglycaemia or rhabdomyolisis, hypoglycaemia starting in childhood, unexplained muscular symptoms (myalgias, high CPK levels), cardiac involvement and/or hepatomegaly.

If there is no suspicion of IEM, fasting of up to 72 h during a hospital stay should be performed in order to rule out insulinoma (incidence of 4/1,000,000 per year). Such a fast enables 99% of hypoglycaemia cases associated with hyperinsulinism to be diagnosed. Blood glucose, cortisol, Growth Hormone, glucagon, insulin, C peptide, proinsulin and beta hydroxybutyrate should be measured every 6 h if the blood glucose level is > 0.6 g/L (with capillary blood glucose test) and then every 1 to 2 h if this level drops below 0.6 g/L. Fasting should be stopped if the blood glucose level is <0.45 g/L and occurs with hypoglycaemia symptoms, or if this level is <0.3 g/L regardless of the symptoms. Baseline blood measurements (with sulfamide and repaglinide) should be performed before glucose intake. A diagnostic work-up to localize the insulinoma should be started only if the fast reveals hyperinsulinemic hypoglycaemia [2,56].

If IEM-related fasting hypoglycaemia is suspected, the following minimal investigations should be performed: a abdominal ultrasound, EKG, echocardiography and laboratory measurements including acylcarnitine profile, total and free carnitin, urine organic acids, CPK, blood lactate levels, triglycerides, uric acid, liver enzymes. Hepatomegaly associated with a high blood lactate level, as well as high triglyceride, is suggestive of glycogenosis. Oral glucose tolerance test has to be performed with measurement of lactates before and 2 h after the glucose intake: a decrease in lactates in the latter situation will suggest type I glycogen storage disease (GSD) whereas an increase in lactates suggests a type III GSD. In both cases, these results should prompt appropriate enzymatic and molecular laboratory investigations. Muscular manifestations associated with a high CPK, unexplained cardiomyopathy, an increase in some forms of acylcarnitine and a carnitine defect should lead to investigation for fatty acid oxidation disorders. In this case, fasting is not mandatory and could be even harmful because of the risk of cardiac arrhythmias, especially in MCAD and VLCAD.

If these minimal investigations are normal, we propose fasting but with close cardiac monitoring: if the blood glucose level is > 0.6 g/L, measurements should be done every 6 h of blood glucose, insulin, C peptide, proinsulin, beta hydroxybutyrate, free fatty acid, blood lactate, total CO2, liver enzymes, alanine and glycerol, and then every 1 to 2 h if this level drops below 0.6 g/L. We propose once daily measurements of CPK, triglycerides, uric acid, urine organic acids and an acylcarnitine profile. Fasting should be stopped if the blood glucose level is <0.45 g/L and occurs with hypoglycaemia symptoms, or if this level is <0.3 g/L regardless of the symptoms. Baseline blood and urine measurements should be performed before glucose intake. If hypoglycaemia does not occur and daily urine organic acid and/or lactate results are not modified, fasting can be continued up to 48 or 72 h with close cardiac monitoring. The orientation of the diagnosis will be based on the results of this fast (Figure 2).

## Conclusion

In adult patients without diabetes mellitus, the diagnosis of hypoglycaemia usually involves classical causes, especially insulinoma. Nevertheless, some hypoglycaemic episodes remain unexplained and less frequent causes should be considered, including inborn errors of metabolism (IEM), particularly in cases of multisystemic involvement. In children, IEM are currently considered a differential diagnosis in cases of hypoglycaemia. In adulthood, IEM-related hypoglycaemia can persist in a previously diagnosed childhood disease. In this case, hypoglycaemic episodes are usually less severe and occur less frequently than in childhood. Hypoglycaemia can sometimes be an indicator of IEM and, although these situations are rare in adults, it is important not to overlook them. A complete physical assessment taking into account the frequent multisystemic involvement, and a few simple laboratory tests (including plasma ammonaemia, lactic acid, acylcarnitine profile and urine organic acid analysis) can orientate the diagnosis towards an IEM. An extensive metabolic and molecular investigation should be performed in all unexplained NIPHS syndromes.

## Abbreviations

ATP, Adenosine triphosphate; BRMCD, Biotin responsive multiple carboxylase deficiency; CACT, Carnitine/acylcarnitine translocase; CDG, Congenital disorders of glycosylation; CPT1, Carnitine palmitoyltransferase-1; CPT2, Carnitine palmitoyltransferase-2; DNA, Deoxyribose nucleic acid; FAO, Defects of fatty acid oxidation; GDH, Glutamate dehydrogenase; GH, Growth Hormone; GSD, Glycogen storage disease; HELLP, Elevated liver enzyme and low platelet count; HFI, Inherited fructose intolerance; HMG-CoA lyase, 3-hydroxy-3-methyl-glutaryl-CoA lyase; HMG-CoA synthase, 3-hydroxy-3-methyl-glutaryl-CoA; HNF 4 alpha, Hepatocyte Nuclear Factor 4 alpha; IEM, Inborn errors of metabolism; LCHAD, Long-chain 3-hydroxyacyl-coA dehydrogenase; MCAD, Medium-chain acyl-coA dehydrogenase; MCT 1, Monocarboxylate transporter 1; MODY, Maturity-onset diabetes of the young; NIPHS, Non-insulinoma pancreatogenic hypoglycaemia syndrome; RH, Reactive Hypoglycaemia; SCHAD, L-3-hydroxyacyl-CoA dehydrogenase; SUR 1, Sulfonylurea receptor 1; UCP2, Mitochondrial uncoupling protein 2; VLCAD, Very long-chain acyl-coA dehydrogenase.

## Competing interests

The authors declare that they have no competing interests.

## Authors’ contributions

CD, MCV and JMS wrote and coordinated the writing of the manuscript; KM, DD and JLW participated in the design of the review and helped to draft the manuscript. All the authors read and approved the final manuscript.
